# Antiseptics in Chronic Dyspepsia

**Published:** 1902-12-20

**Authors:** 


### ANTISEPTICS IN CHRONIC DYSPEPSIA.

In a paper read before the South-west London Medical Society, Dr. Guthrie Rankin [1] shows how much may be done for the relief of dyspepsia by a judicious combination of dietetic and drug treatment, and more especially, so far as the latter is concerned, by the use of antiseptics in combination with the medicines more commonly prescribed. As to the importance of the subject, those with the largest experience will probably most fully agree with Dr. Rankin when he says that as great a depth of long-continued distress may be caused by dyspepsia as by the most deadly diseases, and that there is no other derangement of function to which the human system is liable which can more effectually wreck the happiness of both the individual and those with whom he is brought into daily contact, or more certainly interfere with the quality of the work which he is called upon to perform.

Dr. Rankin deals with his subject under the usual heads of "acute," "atonic," and "nervous" dyspepsia, and lays down some general rules as to the diet and hygiene to be recommended in chronic cases. He adds that where the disorder has been of long standing, success will be more rapidly attained if the stomach is washed out every morning for a few days by the aid of the syphon tube, while in less chronic cases it may suffice to sip a tumblerful of hot water flavoured, if so desired, with a slice of fresh lemon first thing in the morning when the stomach is empty. Of all the antiseptics which he has used, Dr. Guthrie Rankin regards carbolic acid as the best. It is a weak acid, and may be added to either acid or alkaline medicines, two grains of the acid being given in an ounce of the mixture.

In the large majority of cases of atonic dyspepsia early and marked relief is given by the following combination:—Dilute hydrochloric acid, ♏ 15; pure carbolic acid, gr. 2; solution of strychnine, ♏ 5; tincture of ginger, ♏ 20; glycerine, ♏ 30; and water to 1 oz. Later, tonics with carbolic acid may be useful, such as the following in pill or capsule to be taken after meals:—Quinine sulphate, gr. 1; iron sulphate, gr. 1; carbolic acid, gr. $1\frac{1}{2}$; strychnine, gr. $\frac{1}{30}$; extract of cannabis indica, gr. $\frac{1}{4}$. Papain is sometimes useful, and massage and the continuous electric current are of undoubted value.

In acid dyspepsia alkalies are specially indicated. They should be given two or three hours after food, and act best if combined with an antiseptic. A good formula is:—Bismuth carbonate, gr. 30; aromatic spirit of ammonia, ♏ 30 ; carbolic acid, gr. 2 ; solution of strychnine, ♏ 5 ; and (if there be pain) solution of morphine, ♏ 10 ; made into a 1 oz. mixture. Bismuth salicylate in doses of from 10 to 15 grains is also a valuable preparation. When, notwithstanding the use of bismuth the acidity gains the upper hand temporary relief may always be obtained by sipping a wineglassful of very hot water in which 20 or 40 grains of sodium bicarbonate have been dissolved. If the diet consists largely of farinaceous substances two or three ounces of malt infusion, prepared according to Sir William Roberts's formula, should be taken with each meal. It is often of advantage after the hyperacidity is overcome to administer the mineral acids combined as in atonic cases but given *before* food. Later on tonics may be required, still combined with antiseptics.

In nervous dyspepsia it is always desirable to increase the quantity and variety of food as rapidly as possible. Yet, although in these cases the stomach discomfort depends upon nerve exhaustion, fermentation processes take place and much aggravate the evil. Antiseptics therefore have a place here also. A useful formula in this class of cases is :—Zinc valerianate, gr. 3 ; pure carbolic acid, gr. 2 ; arsenious acid, gr. $\frac{1}{40}$ ; extract of cannabis indica, gr. $\frac{1}{4}$ ; in a capsule to be taken after food, three times a day. If sleep be disturbed by gastralgia, sodium bromide, gr. 30, with antipyrin, gr. 15, on going to bed will often give relief.

[1] Brit. Med. Jour., Nov. 29.

